# Intra-articular injection of tranexamic acid via a drain plus drain-clamping to reduce blood loss in cementless total knee arthroplasty

**DOI:** 10.1186/1749-799X-7-32

**Published:** 2012-09-29

**Authors:** Hirotaka Mutsuzaki, Kotaro Ikeda

**Affiliations:** 1 Department of Orthopaedic Surgery, Ibaraki Prefectural University of Health Sciences, 4669-2 Ami, Ami-machi, Inashiki-gun, Ibaraki, 300-0394, Japan; 2 Department of Orthopaedic Surgery, Ichihara Hospital, 3681 Ozone, Tsukuba, Ibaraki, 300-3295, Japan

**Keywords:** Intra-articular injection, Tranexamic acid, Drain-clamping, Blood loss, Cementless, Total knee arthroplasty

## Abstract

**Background:**

Patients undergoing cementless total knee arthroplasty (TKA) sometimes suffer large blood loss. In a retrospective study, we explored whether postoperative intra-articular retrograde injection of tranexamic acid (TA) and leaving a drain clamp in place for 1 h reduced blood loss.

**Patients and methods:**

Patients (*n* = 140) treated with unilateral primary cementless TKA (posterior cruciate ligament retained) were divided into two groups: those who had an intra-articular injection of TA (1000 mg) and drain clamping for 1 h postoperatively (study group, *n* = 70) and those who were not given TA and did not undergo clamping of their drains (control group, *n* = 70). Postoperative total blood loss, volume of drainage, hemoglobin level, transfusion amounts and rates, D-dimer level at postoperative day (POD) 7, and complications were recorded.

**Results:**

Total blood loss, total drainage, mean transfusion volume, and transfusion rates were lower in the study group than in controls (*P* < 0.001). Hemoglobin levels on PODs 1 and 14 were similar in the groups, but on POD 7 the hemoglobin level was higher in the study group than in controls (*P* < 0.001). D-dimer level on POD 7 was lower in the study group than in controls (*P* < 0.05). There were no complications in either group.

**Conclusions:**

Immediately postoperative intra-articular retrograde injection of TA and 1 h of drain-clamping effectively reduced blood loss and blood transfusion after cementless TKA. We believe that this method is simple, easy, and suitable for these patients.

## Introduction

Blood loss is unavoidable after total knee arthroplasty (TKA) [1-3]. Controlling perioperative blood loss is especially a major concern with cementless TKA [4-7]. Two recent meta-analyses were performed to evaluate the use of intravenous tranexamic acid (TA) to reduce blood loss and the need for transfusion after total hip arthroplasty and TKA [8,9]. Based on the results of clinical trials considered suitable for evaluation, it was concluded that TA appears to be a cost-effective, safe method to reduce blood loss, and thus the need for allogenic blood transfusion, without increasing thromboembolic complications.

Tranexamic acid inhibits tissue fibrinolysis for up to 17 h and consequently stabilizes the possibility of clots entering the extravascular space and accumulating in tissues [10]. It is generally accepted, however, that only a small percentage of intravenously injected TA reaches the target location. Thus, a more efficient method (i.e., intra-articular injection) to deliver TA is desirable [11,12]. Another method used to reduce blood loss is drain-clamping [13-18].

One strategy for reducing blood loss after cementless TKA includes drain-clamping combined with intra-articular administration of TA plus carbazochrome sodium sulfonate and fibrin glue [19]. We modified that method to make it simpler, easy to use, suitable for these patients, and understandable for clinicians. We administered TA intra-articularly after cementless TKA, injecting it retrogradely through the drain at the end of the operation and then clamped the drain for 1 h.

The effect of this treatment on reducing postoperative bleeding after cementless TKA has not been reported. Our hypothesis was that intra-articular administration of TA via the drain and then clamping the drain for an hour would reduce postoperative bleeding after cementless TKA.

### Patients and methods

This study, conducted from June 2007 through March 2012, was nonrandomized and retrospective. It included 140 knees undergoing unilateral primary cementless TKA (retaining the posterior cruciate ligament). The ethics committee of Ichihara Hospital reviewed and approved the study (the reference number for the ethics approval: 1202, and the trial registration number: 1202). Informed consent was obtained from each patient. Exclusion criteria were known allergy to TA, cemented TKA, bilateral TKAs, and posterior stabilized TKA. After these exclusions, 140 patients remained. They were divided into two groups. The study group underwent retrograde intra-articular injection of TA via the drain at the end of the operation followed by clamping the drain for 1 h (n = 70) (from January 2010 through March 2012). The control group did not undergo this treatment (n = 70) (from June 2007 through October 2009). The preoperative characteristics, including age, sex, any knee disease, height, weight, preoperative femorotibial angle, range of motion of the knee, and hemoglobin levels 1 day before surgery were comparable in the two groups (Table 1). The backgrounds of the patients in the groups were not significantly different. All surgery was performed or supervised by a single surgeon (K.I.).

**Table 1:** Patient profiles

	**Study group (*n*** **= 70)**	**Control group (*n*** **= 70)**	***P *** **value**
Age (year)	72.0 ± 7.3	74.1 ± 7.1	NS
Sex (male/female)	16/54	17/53	NS
Disease (OA/RA)	68/2	69/1	NS
Height (cm)	150.7 ± 8.0	150.9 ± 7.8	NS
Body weight (kg)	62.3 ± 11.0	63.1 ± 9.5	NS
Preoperative FTA (°)	184.8 ± 6.5	184.7 ± 8.0	NS
Preoperative ROM (°)	108.2 ± 25.4	114.3 ± 18.2	NS
Preoperative Hb (g/dl)	12.6 ± 1.4	12.7 ± 1.4	NS

*OA* osteoarthritis, *RA* rheumatoid arthritis, *FTA* femorotibial angle, *ROM* range of motion, *Hb* hemoglobin.

Results are the mean ± SD.

All patients were given general anesthesia. The patella was not replaced, the posterior curuciate ligament was retained, and all components were fixed without cement in all 140 patients. Surgery was performed under tourniquet control. After a midline skin incision was made, a medial parapatellar approach was used. An intramedullary alignment rod was used for femoral cutting and an extramedullary guide system for tibial cutting. The femoral canal for intramedullary guidance was routinely plugged with bone. We did not use red cell salvaging device.

The implants used in all patients were the Scorpio NRG CR HA (Stryker Howmedica Osteonics, Allendale, NJ, USA) or the NexGen CR (Zimmer, Warsaw, IN, USA). With the NexGen CR type, the femoral component was HA-TCP CR Femoral or CR-flex Porus Femoral. The tibial component was NexGen CR HA-TCP Tibia Peg, NexGen CR HA-TCP Stem Tibia, or NexGen Trabecular Metal Monoblock Tibia. Scorpio NRG CR HA was used in 19 knees in the study group and 37 knees in the control group. NexGen CR type was used in 51 knees in the study group and 33 knees in the control group. NexGen CR HA-TCP Tibia Peg was used in 3 knees in the study group and 15 knees in the control group. NexGen CR HA-TCP Stem Tibia was used in 13 knees in the study group and 18 knees in the control group. NexGen Trabecular Metal Monoblock Tibia was used in 35 knees in the study group and 0 knees in the control group. HA-TCP CR Femoral was used in 21 knees in the study group and 33 knees in the control group. CR-flex Porus Femoral was used in 30 knees in the study group and 0 knees in the control group. The implant types used were not significantly different in the two groups using the Mann–Whitney *U*-test (*P* = 0.591).

Because of the reduced blood loss, the tourniquet was not released until skin closure and application of a compressive dressing [20]. Intraoperative blood loss was negligible in all patients because the tourniquet was not deflated until wound closure. We used an intra-articular drain connected to a vacuum bag (J-VAC suction reservoir 450 ml; Johnson & Johnson K.K., Tokyo, Japan). In the study group, intra-articular administration of TA was injected via the drain retrogradely before releasing of the tourniquet, and the drain was then clamped for 1 h. We based this interval on previous data indicating that 1 h of clamping was effective [17]. Also, 1 h of clamping is simple and understandable for clinicians.

The amount of TA to be injected was chosen to be 1 ampoule (10% Transamin, 10 ml, 1000 mg; Daiichi-Sankyo, Tokyo, Japan). This dose was found acceptable in previous studies [4-8]. Also, a single ampoule is a simple quantity and understandable for clinicians. In both groups in our study, the drains were removed 48 h after the operation.

Following surgery, all patients underwent intravenous prophylactic antibiotic therapy consisting of 1 g cefazolin every 12 h for 3 days. Standard thromboprophylaxis was also prescribed for all patients after surgery. It was administered subcutaneously and included 1.5–2.5 mg fondaparinux sodium (Arixtra® Injection; Glaxo-Smith-Kline, Brentford, Middlesex, UK) starting 24 h after surgery and continuing every 24 h for 10 days. A foot pump (Novamedix A-V Impulse System; Kobayashi Medical, Osaka, Japan) and antiembolic stockings (Ansilk®; ALCARE, Tokyo, Japan) were used for thromboembolic prophylaxis [21,22].

Sutures were removed from all patients 2 weeks after surgery. As part of the postoperative care for both groups, continuous passive movement was started on postoperative day (POD) 3, and standing and full weight-bearing walking were allowed 1 week after the operation.

If possible, autologous blood was collected on preoperative day 4 or earlier. Indications for preoperative autologous blood donations were based on the Standards for Preoperative Autologous Blood Donations (2007) developed by the Japanese Society of Autologous Blood Transfusions. A blood hemoglobin level of ≥ 11.0 g/dl or a hematocrit of ≥ 33% was required. No age limit was established. Iron injection was administered at a dose of 80 mg at blood collection. When collection of ≥ 800 ml of blood took ≥ 1 week, 24,000 units of recombinant human erythropoietin (ESPO®, epoetinum alfa; Kyowa Kirin, Tokyo, Japan) was administered subcutaneously. On POD 1, all the autologous blood was transfused to each patient.

We used the principle of transfusion based on the criteria and guidelines for perioperative transfusion suggested by the National Institutes of Health Consensus Conference, which states that the decision to transfuse blood depends on clinical assessment aided by laboratory data indicating that the patient has symptoms and signs associated with acute anemia [23]. Therefore, our indication for blood transfusion was set at a hemoglobin concentration of 8.5 g/dl or a postoperative hemoglobin level of 8.5–9.0 g/dl with clinical evidence of acute anemia [24]. It may be adjusted according to the patient’s cardiovascular status.

Bleeding-related outcome measures included drained-blood loss, total postoperative blood loss, need for autogenic or allogenic blood transfusions, and the transfusion rate. Hemoglobin levels were measured on PODs 1, 7, and 14. A formula proposed by Nadler et al. [25] and Sehat et al. [26] was used to calculate the total postoperative blood loss. It was based on the maximum postoperative decrease in hemoglobin level adjusted for the weight and height of the patient. The loss of Hb was then estimated according to the following formula:

(1) $$\begin{array}{c} \text{Blood volume}\left(\text{l}\right)=\text{height}{\left(\text{m}\right)}^{3}\text{x}\;0.356\;+\;\text{body weight}(\text{kg})\;\text{x}0.033\;+0.183(\text{woman})\\ =\text{height}{\left(\text{m}\right)}^{3}\text{x}0.367\;+\;\text{body weight}(\text{kg})\text{x}0.032+0.604(\text{man})\\ {\text{Hb}}_{\text{loss}}(\text{g})=\text{Blood volumex}10\text{x}\;({\text{Hb}}_{\text{i}}-{\text{Hb}}_{\text{fin}})+{\text{Hb}}_{\text{t}}\\ \text{Total blood loss}(\text{ml})={\text{Hb}}_{\text{loss}}/{\text{Hb}}_{\text{i}}\text{x}100 \end{array}$$

where Hb_loss_ (g) was the amount of Hb lost, Hb_i_ (g/dl) was the Hb concentration before surgery, Hb_fin_ (g/dl) was the Hb concentration on POD 7, and Hb_t_ (g) was the total amount of allogeneic and autologous Hb transfused.

We recorded the operating time, and the D-dimer level was assayed 7 days after the surgery. We also monitored the wound condition (skin necrosis, hematoma, infection) and the possibility of deep venous thrombosis (DVT) and/or pulmonary embolism (PE) for 4 weeks after the operation.

### Statistical analyses

Student’s *t-*test was used to analyze parametric data, and the Mann–Whitney *U*-test was used for nonparametric data. *P* ≤ 0.05 was considered a significant difference.

## Results

The results are summarized in Table 2. The total blood loss was less in the study group than in the control group (633.8 ± 317.2 vs. 1276.0 ± 327.1 ml, *P* < 0.001). The total drainage during 0–48 h postoperatively was less in the study group than in the control group (271.1 ± 201.8 vs. 506.4 ± 256.8 ml, *P* < 0.001). There was a greater reduction in both autogenic and allogeneic transfusion rates in the study group than in the control group (10.0% vs. 65.7%, *P* < 0.001 and 10.0% vs. 31.4%, *P* < 0.001, respectively). The mean amounts of transfusion per patient for both autogenic and allogeneic transfusions were less in the study group than in the control group (40.0 ± 120.9 vs. 264.1 ± 195.5 ml, *P* < 0.001 and 0.2 ± 0.7 vs. 1.1 ± 1.7 units, *P* < 0.001, respectively). The hemoglobin level on POD 1 was similar in the two groups, but on POD 7 it was greater in the study group than in the control group (10.7 ± 1.3 g/dl vs. 10.1 ± 1.1 g/dl, *P* < 0.001).

**Table 2:** **Postoperative data for allpatients**

**Parameter**	**Study group (*n*** **= 70)**	**Control group (*n*** **= 70)**	***P *** **value**
Operative time (min)	68.8 ± 14.2	82.7 ± 16.8	< 0.001
Postoperative Hb (g/dl)			
POD 1	11.3 ± 1.2	11.1 ± 1.3	NS
POD 7	10.7 ± 1.3	10.1 ± 1.1	< 0.001
POD 14	10.9 ± 1.1	10.7 ± 1.0	NS
Blood loss (ml)			
Drained	271.1 ± 201.8	506.4 ± 256.8	< 0.001
Total	633.8 ± 317.2	1276.0 ± 327.1	< 0.001
Transfusions			
Autologous (ml)	40.0 ± 120.9	264.1 ± 195.5	< 0.001
Allogeneic (units)	0.2 ± 0.7	1.1 ± 1.7	< 0.001
Transfusion rate (%)			
Autologous	10.0	65.7	< 0.001
Allogeneic	10.0	31.4	< 0.001
D-dimer level 7days after surgery (μg/ml)	6.8 ± 2.4	7.7 ± 3.8	0.042

Results are the mean ± SD.

The operating time was shorter in the study group than in the control group (68.8 ± 14.2 vs. 82.7 ± 16.8 min, *P* < 0.001). There were no significant differences in the two groups regarding the implant types. The D-dimer level, measured on POD 7, was lower in the study group than in the control group (6.8 ± 2.4 vs. 7.7 ± 3.8 μg/ml, *P* = 0.042). Finally, symptomatic deep vein thrombosis, pulmonary embolism, and wound trouble including necrosis, infection, and hematoma were not observed in either group.

## Discussion

The most important finding of the present study was that intra-articular retrograde injection of TA via the drain at the end of the operation and then clamping the drain for 1 h effectively reduced postoperative blood loss and thus the need for blood transfusion after cementless TKA. This method is simple, easy to perform, suitable for these patients, and understandable for clinicians. Although, the total blood loss in the controls was greater than that of previous studies [4,5], the use of fondaparinux sodium for thromboprophylaxis can be influenced. The effect of TA and drain-clamping in patients undergoing cementless TKA was certainly clarified, similar to previous studies [11-18].

The fibrinolytic system is activated transiently after any surgery [27]. Tranexamic acid is a synthetic amino acid that inhibits fibrinolysis by reversibly blockading lysine-binding sites on plasminogen molecules, thereby inhibiting its activation. This prevents plasmin from binding with fibrinogen and fibrin structures after clot formation [28]. Because of its antifibrinolytic effects, the risk of increasing venous thromboembolism when using TA is a cause for concern [29,30]. Tranexamic acid does not influence fibrinolytic activity in vein walls, however [30]. Therefore, neither our study nor previous studies observed a greater incidence of venous thrombosis in patients treated with TA [31-33]. Also, the D-dimer level in the study group was lower than that in the control group. The incidence of subcutaneous and intra-joint hematomas was reduced in the study group compared to the control group because of the small amount of bleeding after TKA.

One of the main problems after TKA is the need for blood transfusion. Although the incidence is low, serious complications involving allogeneic blood transfusions (e.g., viral infections, graft-versus-host disease) have been reported [34]. Because the need for blood transfusion was reduced using our method, transfusion-associated complications were reduced. Intra-articular TA application after TKA has recently been introduced and has proved to reduce significantly postoperative blood loss and knee swelling [11,12]. It can also reduce the dose of TA needed to reduce postoperative blood loss [12]. Disadvantages of intravenous TA are prolonged high systemic drug levels from multiple injections or continuous infusion, leading to drug-induced systemic thromboembolic events [8,9]. Further investigations using smaller amounts of TA than were given in this study may be required.

In our study, the operating time was longer for the control group than for the study group. This finding may have been influenced by our use of different implant types. The NexGen Trabecular Metal Monoblock Tibia implant had a trend to be used more often in the study group than in the control group. The tibial base plate of this device originally was fixed with a polyethylene insert. Also, screws were not used for fixation of the tibia to the tibial base plate. Other tibial base plates have screws that fix the tibia and separate the polyethylene insert. Increased time may be required for operations in which screws are used for fixation of the tibia to the base plate and for inserting polyethylene. Although it is unclear whether there was greater blood loss in the control group, a longer operating time does not imply a greater chance of postoperative bleeding [35]. Further investigations using the same implant may be required.

The study has limitations. First, it was retrospective. Although there were no differences in patient characteristics in the two groups, selection bias was not completely excluded. Second, more studies (randomized controlled trials) are needed to determine dosage and administration. Also, investigations using thromboembolism screening tests such as ultrasonography may be required.

Intra-articular injection of TA retrogradely via the drain at the end of the operation and clamping the drain for 1 h effectively reduced postoperative blood loss and the need for blood transfusion after cementless TKA. We believe that our modifications of this technique have made it a simple, easy-to-use method that is suitable for these patients and understandable for clinicians.

## Competing interests

The authors declare that they have no competing interests.

## Authors' contributions

HM and KI conceived of the study and participated in its design and coordination. H.M. participated in the sequence alignment and drafted the manuscript. All authors have read and approved the final manuscript.
